# Supplementary material for: Patterns of Intron Gain and Loss in Fungi
Source: PLoS Biol. 2004 Nov 30;2(12):e422. doi: 10.1371/journal.pbio.0020422 (PMC532390; doi:10.1371/journal.pbio.0020422)
Supplement: Table S1 — Also available at http://genes.mit.edu/NielsenEtAl/. (4.3 MB ZIP). [file pbio.0020422.st001.zip › NielsenEtAl/html/1296.html]

AN6681.1.NCU03039.1.MG00348.1.FG07049.1


```
 CLUSTAL W (1.82) Multiple Sequence Alignments - Introns Inserted


Sequence 1: NCU03039.1	584 aa
Sequence 2: MG00348.1	640 aa
Sequence 3: FG07049.1	564 aa
Sequence 4: AN6681.1	624 aa
Alignment Length: 740 aa
Number Identitical Residues: 239 aa
Alignment Score (without introns) 12057


MG00348.1 	MNGESYSSRD1G~GRHGSSRDHQ0SSRTDREDRRDDRHRDRDRDRGDRDRERDRDRDRDR
NCU03039.1	MNGDTYSSRD1S~GRHGSSRDYP0SSRRDRDDRRD---------RGDRS---------DR
FG07049.1 	MNGDSYSSRD1G~RRGG--RDYP0P-RGDRDDRRD-------RHRGDRD-----------
AN6681.1  	MNGDTYSSR-~G1ADSGRPRDHY0R-----DERRE---------RGDRG-----------
          	***::****  .    * .**:        ::**:         ****.           

MG00348.1 	DRPRRRSRSP-EYRGSRSSRRDAGGEQDSYSSSRSHRDREREDRYGG----ARDRRDRDW
NCU03039.1	GSGRRRSRSPADYRSSRPRRED--GDFDAYSSSRSHRDREREDRYSGRDRRGPDRGDREW
FG07049.1 	---RRRSRSP-DHRSHR--RGE--GDADAYSSSRNHRDREREDRYSGR---ERRGGDREW
AN6681.1  	--ERRRSRSP--HYGSRSSRRE--AEADSYSSSRDYRAREREDRYSSR------RDDRDY
          	   *******  : . *. * :  .: *:*****.:* *******..         **::

MG00348.1 	DRDRG-----------GPSRRREDGDRRGGG------------DRDRGDPHDR--RAGGG
NCU03039.1	DRDRGADRGADRGADRGADRGADRGAERGGRSRRDDDEGGRRRDRDRDVMGDD--RRGGG
FG07049.1 	DRDRGS----------SRRDARRDDDERPSR-------------REREPYDDR--RRGGG
AN6681.1  	DRERG-------------DRRRRDYDDRPSR-----------RERDRDLFEDRPRRDRGD
          	**:**                      * .             .*:*    * . *  *.

MG00348.1 	RRGGERE----ERRRSASPQPKKREPTPDLTDVVPILERKRRLTQWDIKPPGYDSVTAEQ
NCU03039.1	RRDREADR---ERRRSVSPPPKKREPTPDLTDVVPVIERKRRMTQWDIKPPGYGNVTAEQ
FG07049.1 	RERREDRFPAPQERRSASPPPKKREPTPDLTNIVSVLERQRRLTQWDIKPPGYDNVTAEQ
AN6681.1  	RERGGDRR---DRKRSPSP-SRKREPTPDLTDVPSVLTRKRRLTQWDIKPPGYENVTAEQ
          	*.         :.:** ** .:*********:: .:: *:**:********** .*****

MG00348.1 	AKLS-~---GMFPLPGAPRQQTMDPTKMPGGGHRGDRDRDGHRGRDGRDGGRDGRDGGRD
NCU03039.1	AKLS-~---GMFPLPGAPRQQAMDPTKLQAFMTQ--------------------------
FG07049.1 	AKLS-~---GMFPLPGAPRQQPMDPSKLQAFMNQ--------------------------
AN6681.1  	AKLSA1DDLGMFPLPGAPRQQPMDPSRLQAFMNQS-------------------------
          	****: .. ************.***::: .   :.                         

MG00348.1 	GGRDSGRDGDREGGERRGGADSNVTLRPTNSRQSKRLILSNLPAGTTEDSLISFLNLQLN
NCU03039.1	----------------PGGAVNSAALKPTNSRQSKRLIVSNIPPSATDESLLGFFNLQLN
FG07049.1 	----------------PGGQVTSASLKASNSRQSKRLLVSRIPPGTSEDALIAFFNLQLN
AN6681.1  	----------------GGGSADTSALKPSNSRQAKRLFVYNLPPNATVENLVSFFNLQLN
          	                 **   . :*:.:****:***:: .:*..:: : *:.*:*****

MG00348.1 	GLNVIEASDPCLACQMAPDGSFAMVEFRSPSDTTVAYALDGISMEAEDAGNGDANGAASK
NCU03039.1	GLNVIDSADPCVQCQISPDHSFAMLEFRNSPDATVALALDGITMEAEDANG--AAGAG--
FG07049.1 	GLNVIDTTDPCVLCQFSNDRSFAVIEFKDAPETTVALALDGISMEANDASN--GADGGHR
AN6681.1  	GLNVIQSVDPCISAQISDDHSFALLEFKSPNDTTVALALDGITMGEHESNG---ENGAAK
          	*****:: ***: .*:: * ***::**:.. ::*** *****:*  .::..    ...  

MG00348.1 	GLAMRRPKDYIVPAVVDDTGYEPG--VVSSRVVDTPHKISVTNLPAYLTDEQVVELLSSF
NCU03039.1	GLKIRRPKDYIVPAIVEDPNYDPDSEVPSSIVIDSPNKISVTNIPAYLSEEQIMELLVAF
FG07049.1 	GLEIRRPRDYVVPAVTEDVAYDPE--VVSNVVPDTVNKLSITNIPPFLTEEQIIELLASF
AN6681.1  	GLEVRRPKDYIVPNLAEQDLEGAS---GMKDVPDSPNKICVSNIPQYIPEEPVTMLLKSF
          	** :***:**:** :.::    .      . * *: :*:.::*:* ::.:* :  ** :*

MG00348.1 	GELKALVLAKDSSTEESR0GIAFCEYVDVTNTD-VAIEGLNGMELGDKRLKVRKASIGIT
NCU03039.1	GKLKSFVLVKDKHTEESR0GIAFCEYHDSSVTS-VAIDGLNNMMLGDRALKVQKASYGIQ
FG07049.1 	GKPKAFVLVKDRGTEESR0GIAFAEYQDPAVSNPTALDTLNGMDIGGKQIKVSKASIGPT
AN6681.1  	GELKSFVLVKDSSTEESR0GIAFCEYADPNTTT-IAVQGLNGMELGDRHLKVVRASIGMT
          	*: *::**.**  ***** ****.** *   :   *:: **.* :*.: :** :** *  

MG00348.1 	QVSGMEMGVNAMSMLAGTVAQDPDLSPVLQLLNMVTADELMDNDDYEE1ICEDVQEECAK
NCU03039.1	QVAG-ELSVNAMSMLAGTTSLDGDVSRVVQLLNMVTADELMDNDDYEE1IRDDVQEECEK
FG07049.1 	QVANFDVGITAISGLASQTANEVESSRVLQLLNMVTAEELLDNDDYEE1ICEDVREECSK
AN6681.1  	QAAGLDMGVNAMSMFAKTTSQDLESSRVLQLLNMVTPEELMDNEDYEE1ICDDVRDECSK
          	*.:. ::.:.*:* :*  .: : : * *:*******.:**:**:**** * :**::** *

MG00348.1 	YGTVIELKVPRPSSGAKQAAGVGKIYVKFDSIESSTKALKALGGR~KFADR--TVVTTYF
NCU03039.1	FGTIVSLKIPRPTGGSRQSAGVGKIFIKYENSDQATKALKALAGR~KFADR--TVVATYF
FG07049.1 	YGKILDVKVPRPTGGSRQSAGVGKIFVKYEHTEDTTKALQALAGR1PDPERYTLVILATW
AN6681.1  	FGRVLELKIPRPTGGSRQSPGVGKIFVKFETIEATTAALKSLAGR~KFSDR--TVVTTYF
          	:* ::.:*:***:.*::*:.*****::*::  : :* **::*.**   .:*   *: : :

MG00348.1 	PE0EN---~-----------------------FAVNAC2GGMKDSWKVLLKVIVRSSVA-
NCU03039.1	PE0EN---~-----------------------FDVNAW~---------------------
FG07049.1 	TE~GA---~-----------------------GVFHRC~TELFQLKM-------------
AN6681.1  	SE0TIRHP0FTRRIPSSTSVSHETDQGLNCPFALLYWC~CPLFMLTPDRGKDEHPSSTIQ
          	.*     .  :   .:::: : .:... ...   .               .    ::  .

MG00348.1 	------------------------------
NCU03039.1	------------------------------
FG07049.1 	------------------------------
AN6681.1  	YAEVDGTAFAGKVEGKSDSTQPRYIEPPSS
          	 :. ..:: :.. ...:.::..   ...::
```
